# Full-Space Wavefront Shaping of Broadband Vortex Beam with Switchable Terahertz Metasurface Based on Vanadium Dioxide

**DOI:** 10.3390/nano13233023

**Published:** 2023-11-26

**Authors:** Xueying Li, Ying Zhang, Jiuxing Jiang, Yongtao Yao, Xunjun He

**Affiliations:** 1 College of Science, Harbin University of Science and Technology, Harbin 150080, China; 2 National Key Laboratory of Science and Technology on Advanced Composites in Special Environments, Harbin Institute of Technology, Harbin 150001, China; 3 Key Laboratory of Engineering Dielectric and Applications, Ministry of Education, School of Electrical and Electronic Engineering, Harbin University of Science and Technology, Harbin 150080, China

**Keywords:** terahertz metasurface, vortex beam, wavefront shaping, VO_2_, full-space

## Abstract

Currently, vortex beams are extensively utilized in the information transmission and storage of communication systems due to their additional degree of freedom. However, traditional terahertz metasurfaces only focus on the generation of narrowband vortex beams in reflection or transmission mode, which is unbeneficial for practical applications. Here, we propose and design terahertz metasurface unit cells composed of anisotropic *Z*-shaped metal structures, two dielectric layers, and a VO_2_ film layer. By utilizing the Pancharatnam–Berry phase theory, independent control of a full 2π phase over a wide frequency range can be achieved by rotating the unit cell. Moreover, the full-space mode (transmission and reflection) can also be implemented by utilizing the phase transition of VO_2_ film. Based on the convolution operation, three different terahertz metasurfaces are created to generate vortex beams with different wavefronts in full-space, such as deflected vortex beams, focused vortex beams, and non-diffraction vortex beams. Additionally, the divergences of these vortex beams are also analyzed. Therefore, our designed metasurfaces are capable of efficiently shaping the wavefronts of broadband vortex beams in full-space, making them promising applications for long-distance transmission, high integration, and large capacity in 6G terahertz communications.

## 1. Introduction

According to classical Maxwell’s theory, it is well known that the angular momentum of the electromagnetic wave generally comprises two principal components: spin angular momentum (SAM) and orbital angular momentum (OAM). The SAM is associated with the polarization state of the electromagnetic wave, while the OAM is linked to its spatial phase distribution [1,2,3]. Unlike the SAM carrying two discrete states per photon (±*ћ*), the OAM can be expressed as *lћ*, where *l* denotes the topological charge and can theoretically be an unbounded integer. Recently, the investigation of OAM has emerged as a prominent topic of research and played an important role in the field of modern communications [4]. As one specific form of OAM, fortunately, the vortex beams with a spiral phase have also attracted considerable attention from researchers [5]. Additionally, it is noteworthy that the vortex beams with distinct OAM modes are orthogonal to each other, leading to their excellent isolation. This unique property dramatically increases the channel capacity and spectral efficiency within a single communication channel through the OAM multiplexing. Currently, various methods have been employed to generate vortex beams with diverse topological charges [6]. For instance, a polyethylene spiral phase plate has been developed to generate a vortex beam by introducing different transmission phase delays [7]. Circular antenna arrays with uniform amplitude but different phases have been explored for OAM-multiplexed vortex beam communications [8]. Moreover, single-patch antennae have been used to generate the vortex beams by modifying the patch shape and feeding structures [9]. Despite notable progress in the vortex beam generation, traditional methods still suffer from intrinsic limitations, such as bulky dimensions and complex feeding networks. Consequently, these constraints greatly impede their practical applications. Therefore, there is a significant need for more advanced vortex beam generators that can meet the requirements of high integration and large channel capacity in 6G terahertz communications. 

Metasurfaces, 2D ultrathin counterparts of metamaterials, are composed of engineered subwavelength meta-atoms that are arranged by periodic or quasi-periodic arrangements [10]. These metasurfaces have the unexpected ability to manipulate the amplitude, phase, and polarization of electromagnetic waves. Through the appropriate engineering of the sizes and arrangements of artificial meta-atoms, different types of metasurfaces, spanning from microwaves to visible lights, have been demonstrated to implement versatile functionalities, including anomalous refraction [11,12], focusing [13,14], holography imaging [15,16], and Bessel beams [17,18], to name a few. Benefiting from their ultrafine nature and unparalleled control over electromagnetic waves, this allows metasurfaces to serve as a novel platform for the generation of terahertz vortex beams and the realization of versatile applications involving these beams [19,20]. By and large, such metasurfaces can be categorized into resonance-based metasurfaces and geometric-phase metasurfaces, based on their phase responses. For example, Zhang’s group proposed ultrathin vortex phase plates based on a complementary V-shaped antenna structure to produce a terahertz vortex beam with a topological number *l* = 1 [21]. R. Dharmavarapu et al. designed a polarization-insensitivity metasurface composed of a dielectric cross-shaped resonator array to generate a vortex beam with a topological charge *l* = 1 at 0.78 THz [22]. Furthermore, VO_2_-based reflective meta-atoms are also used to generate a dynamically switchable focusing vortex beam by tailoring their conductivity [23]. However, these investigations mainly focus on creating a single narrowband terahertz vortex beam in either reflection or transmission mode. If a specific shaped vortex beam is required, additional components need to be added to the optical path, leading to complicated terahertz systems [24]. Compared to the pure generation of vortex beams in either transmission or reflection space, moreover, full-space wavefront shaping and working bandwidth of the vortex beam generators based on terahertz metasurfaces have been poorly reported so far [25]. Therefore, designs and realizations of full-space wavefront shaping of broadband terahertz vortex beams are highly urgent and indispensable for practical applications. 

**Figure 1:**
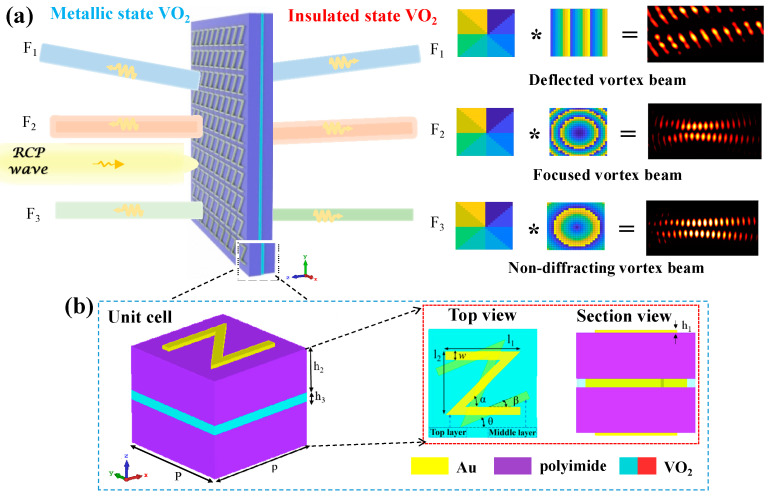
Schematic diagram of terahertz metasurfaces working in full-space: (**a**) functional illumination of metasurfaces; (**b**) structure and parameters of metasurface unit cell.

To address the aforementioned issues, this paper focuses on improving the shaping, bandwidth, and generation space of vortex beams. As a solution, we propose and design a multilayer metasurface unit cell composed of three anisotropic *Z*-shaped metal structures, two dielectric layers, and a phase change material layer. One key advantage of this design is the ability to actively switch working modes between transmission and reflection thanks to the phase transition characteristics of the phase change material. By carefully adjusting the spatial orientations (or orientation angles) of the anisotropic Z-shaped structures, moreover, independent control of a full 2π phase coverage within a wide terahertz range can be achieved based on the Pancharatnam–Berry phase theory. To validate our concept, we constructed three broadband multilayer metasurfaces using the unit cell array described above to generate a full-space deflected vortex beam (F_1_), a full-space focused vortex beam (F_2_), and a full-space non-diffraction vortex beam (F_3_), respectively, as shown in Figure 1. Therefore, our designed multilayer metasurfaces would exhibit great potential for applications in 6G terahertz communications, specifically in terms of high integration and large capacity. 

## 2. Design and Simulation of Metasurface Unit Cells

Figure 1 shows the schematic illustration of the proposed multilayer metasurfaces for generating terahertz vortex beams with various shaped wavefront. The unit cell of the proposed metasurface is composed of three-layer same *Z*-shaped metal structures, two dielectric spacer layers, and a phase change material layer, respectively, as displayed in Figure 1b. In this structure, the relative rotation angle between middle metal structure and two adjacent metal structures is *β* = 22.5° to beneficially increase transmission intensity. Moreover, the top and bottom metal structures are deposited on the dielectric, respectively, while the middle metal structure is embedded into the phase-change material layer to reduce the thickness of unit cells. Such structure allows for active full-space control with the phase-change material. Compared with previously reported structures, our unit cell exhibits strong electric and magnetic dipoles due to the introduction of multilayer metal elements, facilitating the creating of a Huygens’s surface [26]. In addition, a full 2π phase coverage can be achieved in a broad terahertz range by only utilizing the Pancharatnam–Berry phase of the unit cells. 

To verify the aforementioned designed functions, the full-wave simulations are conducted by using a finite-difference time-domain (FDTD) solver in commercially available numerical software, CST microwave studio [27,28]. In the simulations, moreover, a tetrahedral mesh with the adaptive mesh scheme is utilized to optimize the numerical simulation accuracy, in which the moderate mesh accuracy is adopted to make good tradeoff between accuracy, memory requirements and simulation time. The right-handed circularly polarized (RCP) terahertz wave illuminates normally on the surface of the designed metasurface unit cell along the *z*-direction. The unit cell boundaries in the *x*- and *y*-directions are set as the periodic boundary, as depicted in Figure 1a. In the numerical calculations, the basic size parameters of the unit cell are optimized by the CST and determined as follows: P = 26 μm; *l*_1_ = 18 μm; *l_2_* = 15.17 μm; *w* = 2 μm; α = 45°; *β* = 22.5°; *h*_1_ = 0.2 μm; *h*_2_ = 10 μm; and *h*_3_ = 2 μm. The gold film with a conductivity of 4.56 × 10^7^ S/m is patterned to create the *Z*-shaped anisotropic metal structures [29], and the lossless polyimide with the permittivity of *ε*PI = 3.5 is selected as the dielectric layer material [30]. 

**Figure 2:**
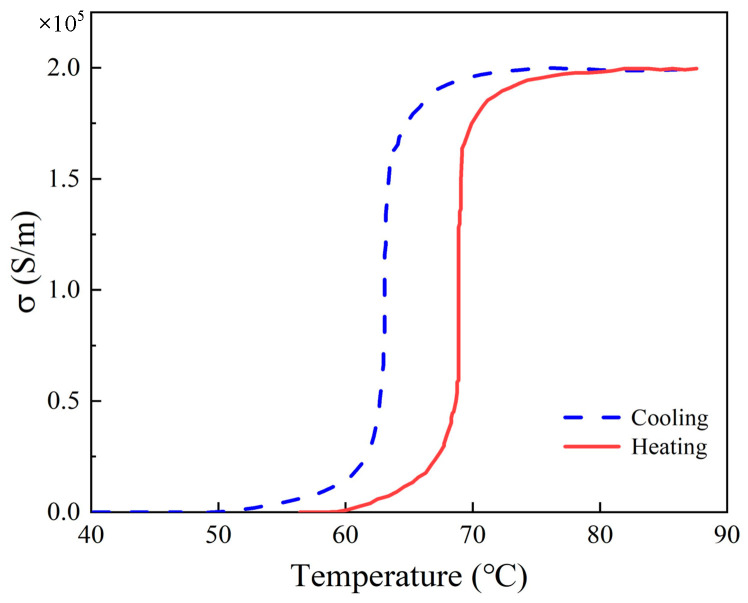
The change in conductivity of VO_2_ at different temperatures.

VO_2_ is adopted as the phase change material due to its adjustable characteristics. Its state transition can be induced by electrical stimulation or light stimulation, and their corresponding response of VO_2_ state transition are 650 *fs* [31] and 200 *fs* [32,33], respectively. Such quick response can meet the fast switching requirement of metasurface in modern 6G technology. By applying external excitations, the VO_2_ can undergo a phase transition from an insulating state to a metallic state, leading to the change in its conductivity by orders. For example, its dielectric constant *ε*_eff_ as a function of temperature can be described by the Bruggeman model [34]:

$$\varepsilon_{eff}=\left\{\varepsilon_{i}\left(2-3F_{m}\right)+\varepsilon_{m}\left(3F_{m}-1\right)+\sqrt{\left[\varepsilon_{i}\left(2-3F_{m}\right)+\varepsilon_{m}{\left(3F_{m}-1\right)}^{2}+8\varepsilon_{m}\varepsilon_{i}\right]}\right\},$$

where *F_m_* is the volume fraction, *ε_i_* and *ε_m_* is the permittivity of VO_2_ in insulating and metallic phases, respectively. Here, *ε_i_* = 9 and *ε_m_* is described by Drude model: $\varepsilon_{m}(\omega )=\varepsilon_{\infty }-\frac{\omega_{P}^{2}}{\omega^{2}+i\omega \gamma }$, with the epsilon infinity *ε*_∞_ = 12, the collision frequency γ = 5.75 × 10^13^ Hz, and the plasma frequency *ω*_p_ = *Ne*^2^/*ε*_0_ m*, in which the effective mass m* = 2*m*_e_, *m*_e_ is the mass of the free electron, and the carrier density is *N* = 8.7 × 10^21^ cm^−3^ [35]. The volume fraction *F*_m_ is a function of surrounding temperature *T*, which is calculated by the Boltzmann function: $F_{m}\left(T\right)=F_{max}(1-\frac{1}{1+exp\left[(T-T_{0})/∆T\right]})$, where *F*_max_ = 0.95 is the maximum value of *F*_m_. *T*_0_ is the phase change temperature, and Δ*T* is the thermal hysteresis temperature. Thus, the conductivity of VO_2_ at different temperatures can be expressed as $\sigma =-i\varepsilon_{0}\omega \left(\varepsilon_{eff}-1\right)$, where *ε*_0_ is the dielectric constant of the vacuum, respectively. *ε* is the dielectric function of the composite system [36]. As *T*_0_ = 154 K and Δ*T* = 2 K at temperature rise, *T*_0_ = 143.6 K and Δ*T* = 6 K at temperature decrease [37]. The variation trend of the conductivity of VO_2_ with temperature is shown in Figure 2. In our work, the conductivity of VO_2_ is 200 S/m at the insulating state, while *σ* = 2 × 10^5^ S/m at the metallic state, approximately corresponding to *T*_c_ = 300 K and *T*_h_ = 400 K, respectively [38].

**Figure 3:**
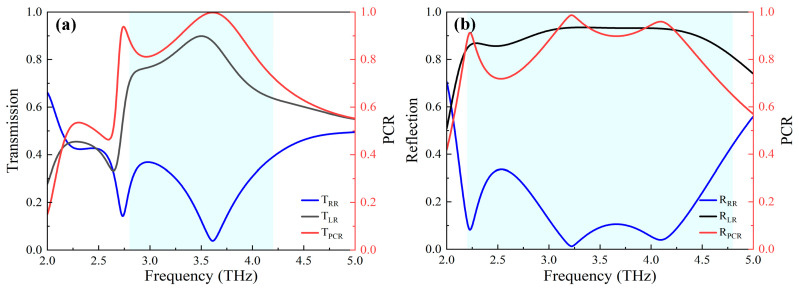
Terahertz responses of the designed unit cell under the RCP incident waves: (**a**) PCR and transmission amplitudes of the cross-polarized and co-polarized output waves; (**b**) PCR and reflection amplitudes of the cross-polarized and co-polarized output waves.

Figure 3 shows terahertz response of the unit cell under the RCP illumination. As expected, the anisotropy of unit cells produces significant circular polarization conversions with a broadband range in full-space (shallow blue region as shown in Figure 3). At the insulating state of VO_2_, moreover, the maximum intensity of cross-polarized transmission exceeds 90% at 3.6 THz, while the co-polarized intensity remains below 4%, thus, obtaining a transmission polarization conversion rate (PCR) of 100%, as shown in Figure 3a. At the metallic state, the cross-polarized and co-polarized reflection intensities are 93% and 1% at 3.2 THz, respectively, resulting in a reflection PCR of 99.8%, as shown in Figure 3b. These results imply that our structures enable effective energy transformation from RCP to LCP in full-space [39]. 

**Figure 4:**
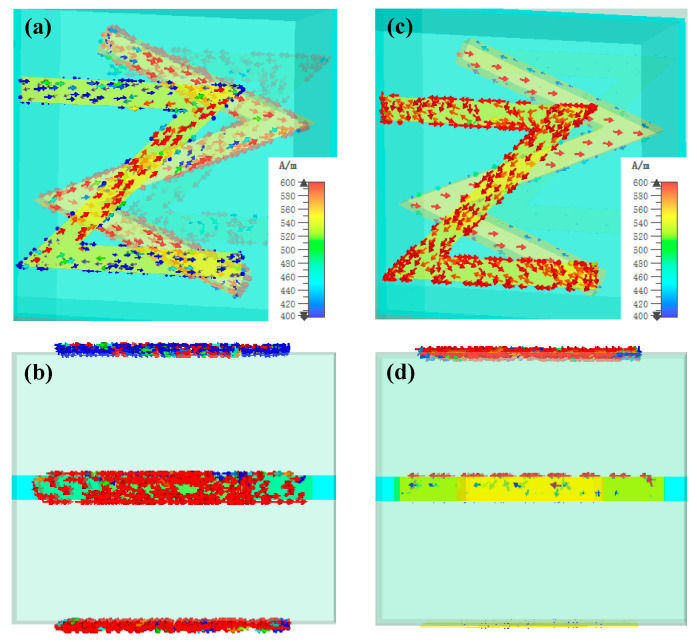
Surface current distributions of the unit cell for different states of VO_2_ under RCP incident wave: (**a**) top view and (**b**) sectional view at the insulating state; (**c**) top view and (**d**) sectional view at the metallic state.

To further discover the working principle and mode of the Z-shaped unit cell, next, we calculate the field distributions of unit cell structure at both the transmission and reflection resonances. The corresponding simulated results are shown in Figure 4. It is observed that at the insulating state, strong surface currents are induced on all three-layer Z-shaped metal structures at 3.6 THz, and here, the Z-shaped unit cell works in the transmission mode. The surface currents on these structures are found to be opposite to each other, signifying the presence of strong magnetic dipoles among them. Additionally, the surface currents on the bottom-layer are distinctly stronger than those on the other layers, suggesting the existence of a net electric dipole, as shown in Figure 4a,b. These induced electric and magnetic resonances interact with each other and can be used to create a Huygens’s surface. As a result, this strong interaction results in a high conversion efficiency from the RCP incident wave to the LCP output wave, as well as a broad working bandwidth. In contrast, at the metallic state, the surface currents mainly focus on the top-layer and middle-layer structures at 3.2 THz, as shown in Figure 4c,d. Here, the incident wave is fully reflected by the VO_2_ layer due to its high conductivity, and the surface currents on the bottom-layer metallic structure are completely suppressed. Thus, the Z-shaped unit cell works in the reflection mode. Therefore, the working mode of the unit cell can be actively switched between transmission and reflection by changing the conductivity of the VO_2_. 

**Figure 5:**
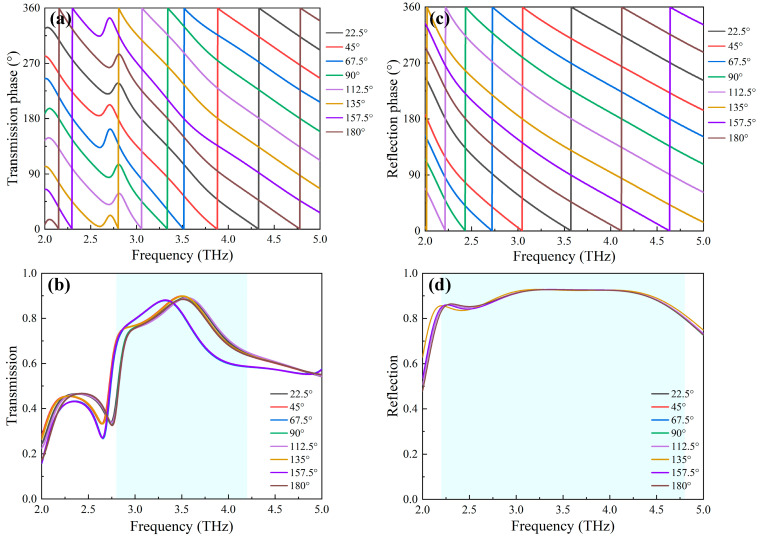
Amplitude and phase of the cross-polarized output wave under different rotation angle *θ*: (**a**) transmission intensity; (**b**) transmission phase; (**c**) reflection intensity; and (**d**) reflection phase.

To facilitate the design of unit cells, the phase response for our unit cell depends only on the spatial rotation of the metallic structures, described by the rotational angle *θ* (as displayed in Figure 1). While the top metal rotates *θ* degrees, the middle layer and the bottom metal rotate by the same angle. As shown in Figure 5, it is observed that based on the Pancharatnam-Berry phase principle, successive phase modulation of 360° for the cross-polarized component is obtained over a wide frequency range, simply by adjusting the rotation angle *θ* from 0 to 180°. For example, when the metallic element is rotated from 22.5° to 180° with a step of 22.5°, the transmission and reflection intensities of the cross-polarized component remain almost unvaried in the respective interested frequency ranges under RCP incident wave illuminations, while their phase responses exhibit a full 2π phase coverage, as shown in Figure 5a–d. Moreover, the suitable linear relation between the phase and rotational angle *θ* is also demonstrated over a broad frequency range due to parallel phase curved lines. Therefore, these excellent properties can be ascribed to the Pancharatnam-Berry phase modulation capability. 

## 3. Result and Discussions

**Figure 6:**
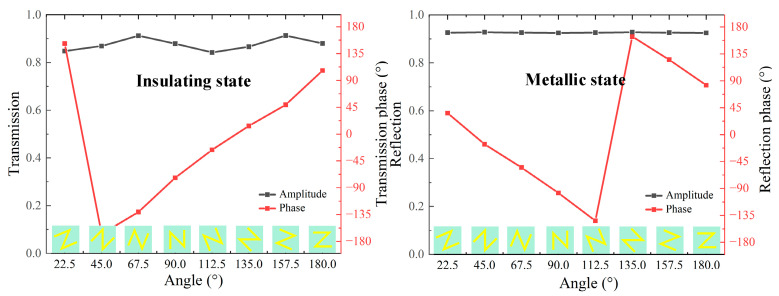
Amplitudes and phases of the cross-polarized wave for eight unit cells at different working modes: (**left**) transmission intensity and phase at 3.6 THz for insulating state; (**right**) reflection intensity and phase at 3.2 THz for the metallic state.

Based on the above analysis, a library of unit cells comprising eight unit cells is created for both reflection and transmission modes. This is achieved by appropriately rotating the spatial direction of the unit cells. As shown in Figure 6, the average cross-polarized transmission and reflection amplitudes of 8 unit cells are above 0.86 at 3.6 and 0.92 at 3.2 THz, respectively. The corresponding transmission and reflection phases exhibit an opposite linear variation step of 45° in a full 2π phase coverage. This fulfills the required condition for independent phase manipulation in both transmission and reflection modes [40]. In the light of the convolution theorem [41], it becomes possible to arbitrarily reshape the wavefront of the vortex beam by deliberately designing different metasurfaces composed of the aforementioned 8-unit cell array. 

### 3.1. Deflected Vortex Beams in Full-Space

**Figure 7:**
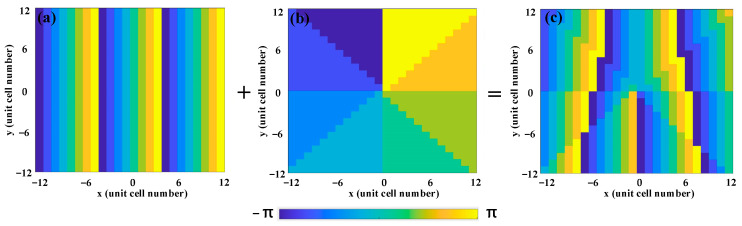
Convolutional phase process of the deflected vortex beam at insulating state under RCP wave illumination: (**a**) phase distribution of the deflected beam along the negative *x*-axis; (**b**) phase distribution of the vortex beam with *l* = 1; and (**c**) phase distribution of the vortex beam with *l* = 1 deflected along the negative *x*-axis.

The conventional method for incorporating the helical mode into the wavefront of a deflect beam typically requires a series of bulky optical components. This not only increases the size and complexity of terahertz systems but also hinders the integration of terahertz devices. To generate a deflected vortex beam, schematically shown in Figure 1a, the functionalities of conventional deflectors and spiral phase plates are integrated into a single metasurface by adopting the convolutional operations. As a result, the phase distributions of this metasurface consist of a deflected linear gradient phase profile $\varphi_{D}$ and a spiral phase profile $\varphi_{V}$, which can be expressed as follows [42]:

(1) $$\psi_{D-V}(x,y)=\varphi_{D}+\varphi_{V\;}=2\pi (sin\theta_{D}\cdot x)/\lambda +l\cdot arctan(y/x),$$

(2) $$\theta_{D}=acrsin(\lambda /\Gamma_{D}),$$

where *λ* represents the wavelength of the incident wave, *l* denotes the topological charge of the vortex beam, $\psi_{D-V}(x,y)$ is the phase of the metasurface, $\theta_{D}$ is the deflected angle of the vortex beam, and Γ is the period of the unit cell. Here, the deflected angle and topological charge are set to be $\theta_{D}$= 24° and *l* = 1 at 3.6 THz for the insulating state, respectively, while they are set to be $\theta_{D}$= 27° and *l* = −1 at 3.2 THz for the metallic state. As a result, we can employ the above two equations to calculate the phase distribution of the deflected vortex beam at an insulating state, as displayed in Figure 7. Figure 7a illustrates the phase distribution of the deflected beam along the negative *x-*axis, and Figure 7b shows the phase distribution of the vortex beam with a topological charge *l* = 1. Additionally, Figure 7c demonstrates the phase distribution after two-phase convolution, indicating the generation of a single deflected vortex beam along the negative *x-*axis. 

**Figure 8:**
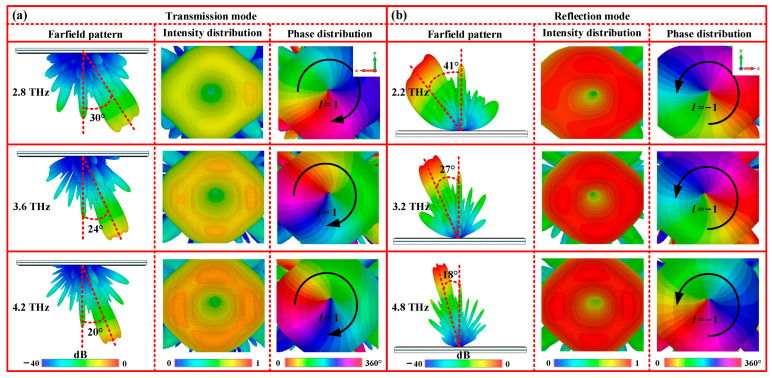
3D far-field radiation patterns of the deflected vortex beam at different working states: (**a**) insulating state (transmission mode); (**b**) metallic state (reflection mode).

To further demonstrate the generation of the deflected vortex beam, a metasurface composed of a 24 × 24 unit cell array occupying an area of 0.624 × 0.624 mm^2^ is designed according to the above-calculated phase distribution. Next, the full-wave simulations are carried out using the FDTD method to study the wavefront of the designed vortex metasurface, as shown in Figure 8. From Figure 8a, it is observed that under RCP wave illuminations, a transmission beam deflects towards the positive *x-*axis at 3.6 THz. The corresponding deflected angle is approximately 24°, which is in agreement with the calculated angle. Moreover, the 2D field and phase distributions reveal two typical features, such as a 2π spiral phase distribution increasing clockwise and a doughnut-shaped intensity distribution. These characteristics align with the properties of the vortex beam with a topological charge of *l* = 1. Therefore, these simulated results demonstrate the successful production of a deflected transmission vortex beam with *l* = 1 by the designed metasurface. However, VO_2_ transitions to a metallic state, and a similar reflection vortex beam deflected towards the negative x-axis is also observed at 3.2 THz, accompanied by a 2π spiral phase variation increasing counterclockwise. This results in the generation of a deflected reflection vortex beam with a topological charge of *l* = −1, as shown in Figure 8b. To verify the broadband performance of the deflected vortex beams, we also calculate the far-field radiation of the transmission and the reflection modes at other frequencies. The numerical simulation results display that similar deflected vortex beams are generated at frequencies of 2.8 THz and 4.2 THz for the insulating state, and frequencies of 2.2 THz and 4.8 THz for the metallic state. The corresponding working bandwidths are 1.4 THz and 2.6 THz, respectively, as shown in the first and third rows of Figure 8a,b. Additionally, it is worth noting that the width of the vortex beam is gradually reduced as the frequency increases, which aligns with the previous report [43]. In short, these results demonstrate that our vortex beams not only operate effectively within a wide frequency range, but also exhibit full-space deflection through the phase transition of VO_2_.

### 3.2. Focused Vortex Beams in Full-Space

**Figure 9:**
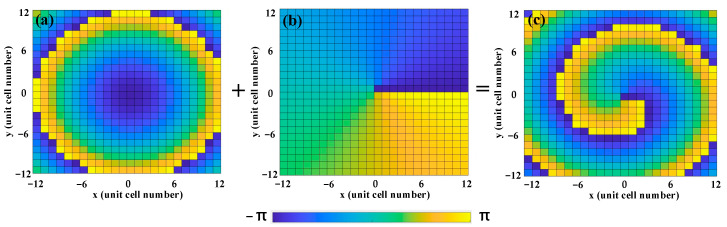
Convolutional phase process of the focused vortex beam at insulating state under RCP wave illumination: (**a**) phase distribution of the focused beam with *F* = 300 μm; (**b**) phase distribution of the vortex beam with *l* = 1; and (**c**) phase distribution of the focused vortex beam with *F* = 300 μm and with *l* = 1.

The traditional vortex beams exhibit significant diffraction phenomena during propagation, resulting in an increase in the divergence angle as the distance increases. This posed a disadvantage for signal-receiving ends in the communication fields [44]. To mitigate the divergence characteristics and enhance the information transmission capabilities, it is necessary to control the energy distribution of the vortex beam along its propagation path. Currently, a widely adopted technique is to use metasurfaces to focus the dispersion of the vortex beam at a specific position, effectively increasing the power intensity of the vortex beams [42]. To generate a focused vortex beam schematically shown in Figure 1a, conventional focused lenses and spiral phase plates are combined into a single metasurface using a convolutional operation. As a result, the phase distributions of this metasurface include the focused phase profile $\varphi_{F}$ and the spiral phase profile $\varphi_{V}$, represented as follows [45]:

(3) $$\psi_{F-V}(x,y)=\varphi_{F}+\varphi_{V\;}=2\pi (\sqrt{{(x}^{2}+y^{2})+F^{2}}-F)/\lambda +l\cdot arctan(y/x),$$

where *F* represents the focal length. In calculation, the focal length and topological charge are set as *F* = 500 μm and *l* = 1 at 3.6 THz for the insulating state, respectively. Similarly, for the metallic state, *F* = 500 μm and *l* = −1 at 3.2 THz are considered. Therefore, the convolution operation is applied to calculate the theoretical phase profiles of the focused vortex beam at the insulating state, as shown in Figure 9. 

**Figure 10:**
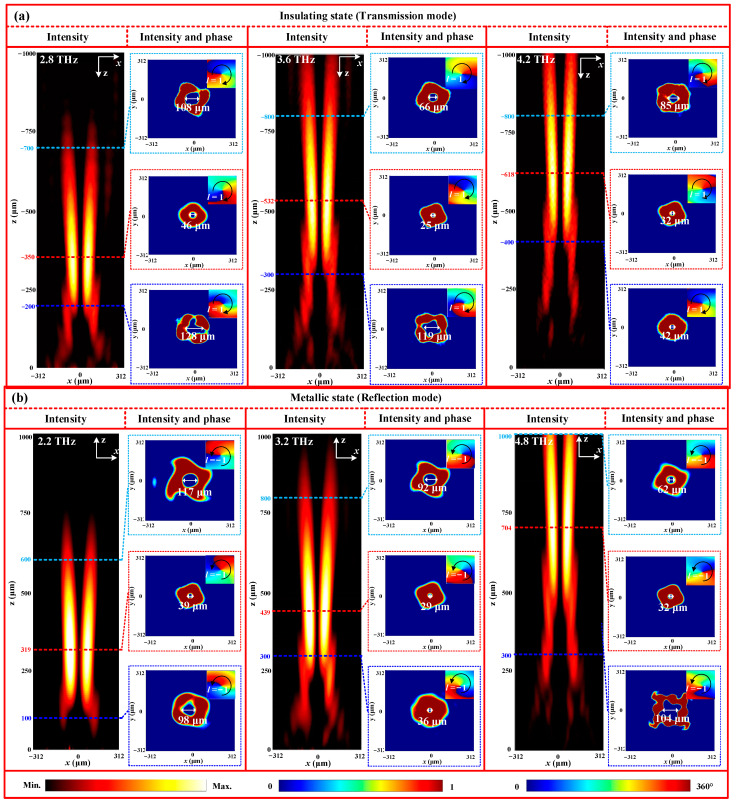
Energy and phase distributions of the focused vortex beam at different working states: (**a**) insulating state (transmission mode); (**b**) metallic state (reflection mode).

To demonstrate the validity of the above calculation, a metasurface, composed of a 24 × 24 unit cell array with a size of 0.624 × 0.624 mm^2^, is constructed to produce a focused vortex beam, as depicted in Figure 10a. From Figure 10a, it can be observed that in comparison to the pure divergence vortex beams, the transmission cross-polarized energy gradually shifts closer to the central axis along the propagation path. At the focal plane, the energy distribution is closest to the central axis before moving away again. This energy distribution reveals the evolution of the wavefront shape of the focused beam. To further verify the vortex characteristics of the focused beam, the 2D intensity and phase patterns at three different *xoy* planes are extracted and presented on the right side of every column of Figure 10a. It is evident that at 3.6 THz, all intensity patterns at the three planes exhibit clear doughnut-shaped energy rings. Additionally, the corresponding phase patterns display a clockwise 2π twist phase wavefront. These key characteristics demonstrate the generation of a vortex beam with a topological charge of 1. Moreover, the inner diameters of the three energy rings are 129 μm, 25 μm and 66 μm for z = −300 μm, z = −532 μm, and z = −800 μm, respectively. These results further imply that the vortex beam possesses the special feature of a focused lens with a focal length of 500μm. Meanwhile, it is important to note that there is a slight discrepancy in the focal lengths between the simulated and theoretical results. This discrepancy can primarily be attributed to the limited number of unit cells (24 × 24) used in the numerical simulation. However, in the metallic state, similar focused vortex beams are also generated at 3.2 THz, as shown in Figure 10b. Additionally, similar results are also observed at 2.8 THz and 4.2 THz for the insulating state, and at 2.2 THz and 4.8 THz for the metallic state, verifying the broadband nature of the focused vortex beam in full-space. 

### 3.3. Non-Diffraction Vortex Beams in Full-Space

**Figure 11:**
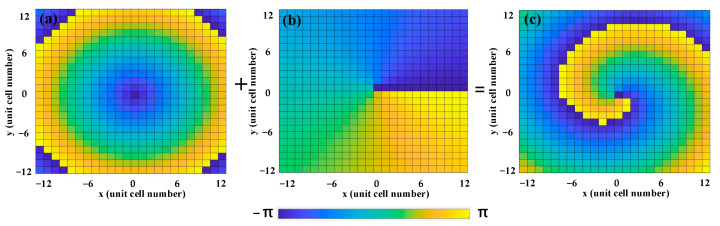
Superposition phase distribution of the non-diffraction vortex beam at insulating state under RCP wave illumination: (**a**) phase distribution of the zero-order Bessel beam with $\theta_{B}$ = 22°; (**b**) phase distribution of the vortex beam with *l* = 1; and (**c**) phase distribution of the non-diffraction vortex beam with *l* = 1 and $\theta_{B}$ = 22°.

Although the obtained focused vortex beams can somewhat reduce their divergence characteristics, the generation of spatially stable field distributions along the propagation direction remains an urgent issue in long-range wireless transmission. To address this limitation, the combination of the Bessel beam and the vortex beam can be employed to create a breakthrough in the unstable nature of the focused vortex beam, thereby significantly enhancing the information transmission capacity of the far-field OAM system. To generate a non-diffraction vortex beam, schematically shown in Figure 1a, here, the function of a focused lens and an axicon lens generating the zero-order Bessel beam is integrated into a single metasurface. By utilizing the convolution operating principle of the Bessel beam phase ($\varphi_{B}$) and spiral phase ($\varphi_{V}$), the corresponding phase distributions can be derived and expressed as follows [46]:

(4) $$\psi_{B-V}(x,y)=\varphi_{B}+\varphi_{V\;}=\frac{2\pi }{\lambda }\sqrt{x^{2}+y^{2}}sin\theta_{B}+l\cdot \arctan\left(y/x\right),$$

where $\theta_{B}$ is the base angle of the zero-order Bessel beam. Figure 11 shows the calculated superposition phase of the vortex beam (*l* = 1) and Bessel beam ($\theta_{B}$ = 22°) at 3.6 THz for the insulating state. 

**Figure 12:**
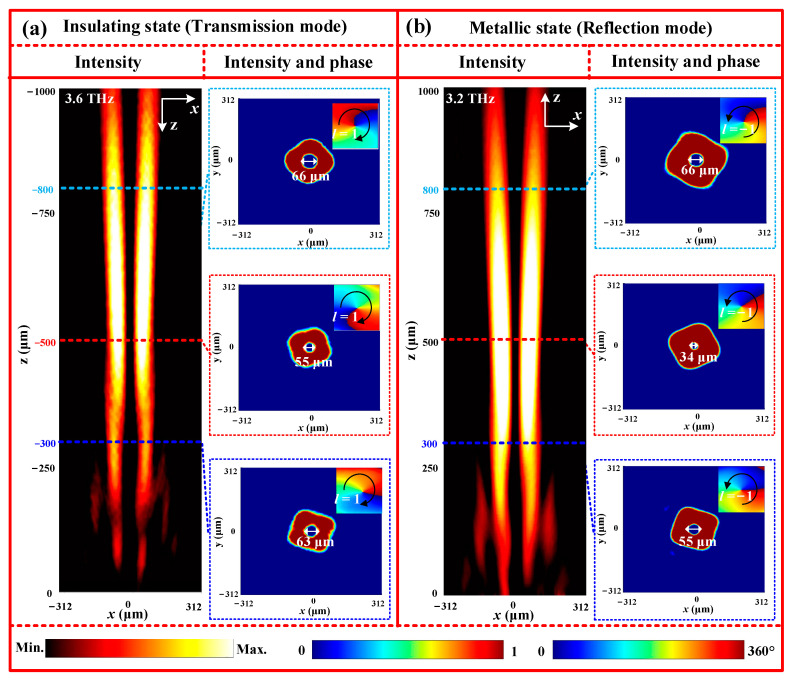
Energy and phase distributions of the non-diffractive vortex beam under different working states: (**a**) insulating state (transmission mode); (**b**) metallic state (reflection mode).

According to this superposition phase mentioned earlier, a metasurface is designed to create a transmission non-diffractive vortex beam with *l* = 1 and $\theta_{B}$ = 22°. The electromagnetic characteristics of this beam are then calculated using the FDTD method, and the simulated energy and phase distributions at 3.6 THz are displayed in Figure 12a. In the first column of Figure 12a, it can be seen that the energy distribution in the *xoz* plane largely concentrates along the center axis, allowing for non-diffractive propagation over a distance of more than 1000 μm. To further identify the vortex nature of the non-diffractive beam, the energy and phase distributions in the different *xoy* planes are presented in the second columns of Figure 12a. Upon analysis, it becomes apparent that the energy intensity at the center is zero, forming a doughnut-shaped energy ring. Additionally, the phase profile exhibits a 2π spiral phase variation in a counterclockwise direction. These characteristics affirm that the transmitted non-diffractive beam is indeed a vortex beam with *l* = 1, which is in line with theoretical expectations. Furthermore, the inner diameters of the doughnut-shaped energy rings in the different transverse planes, namely, z = 300 μm, z = 500 μm, and z = 800 μm, are around 63 μm, 55 μm, and 66 μm, respectively. These values display a slight deviation, implying that the vortex beam maintains centralized propagation with minimal diffraction. In the metallic state of VO_2_, however, a non-diffractive reflection vortex beam with *l* = −1 and $\theta_{B}$ = 22° can be obtained at 3.2 THz, as shown in Figure 12b. In addition, similar results are also observed at 2.8THz and 4.2 THz for the insulating state, and at 2.2 THz and 4.8 THz for the metallic state, verifying the broadband nature of the non-diffractive vortex beam in full-space. Based on these simulation results, therefore, it can be deduced that the designed metasurface is capable of generating various non-diffractive vortex beams in full-space. 

### 3.4. Divergence Characteristics of Different Types of Vortex Beams

**Figure 13:**
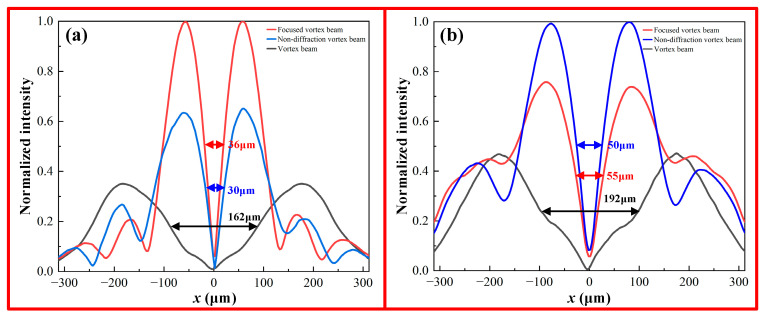
Normalized reflection energy intensity of the vortex beam, focused vortex beam, and non-diffracting vortex beam in different *xoy* planes: (**a**) z = 500 μm; (**b**) z = 800 μm.

To further compare the divergence characteristics of different types of vortex beams, the divergences of the vortex beam, focused vortex beam, and non-diffracting vortex beam are numerically simulated and analyzed. Figure 13 shows the normalized reflection energy distributions of the three different types of vortex beams in xoy planes at z = 500 μm and 800 μm. Here, the bandwidth at half-intensity is used to describe the divergence of the vortex beams. It can be observed that compared with the other two vortex beams, the non-diffracting vortex beam presents excellent convergence upon propagation due to a small half-intensity bandwidth. Beyond the non-diffracting length, however, the non-diffracting vortex beam starts to present a slight divergence, leading to a 20 μm increase in the half-intensity bandwidth. For instance, the half-intensity bandwidths of the vortex beam, focused vortex beam, and non-diffracting vortex beam at z = 500 μm are 162 μm, 36 μm, and 30 μm, respectively. At z = 800 μm, these values increase to 192 μm, 55 μm, and 50 μm, respectively. Therefore, these results indicate that the non-diffracting vortex beam could maintain a high information transmission capacity over long-range propagation. 

## 4. Conclusions

In summary, we have successfully generated broadband deflected vortex beams, focused vortex beams, and non-diffracting vortex beams using various terahertz metasurfaces. These metasurfaces are the array of unit cells composed of anisotropic Z-shaped metal structures, two dielectric layers, and a VO_2_ film layer. Furthermore, by tuning the conductivity of VO_2_, the operational working modes of these vortex beam generators can be dynamically switched between transmission and reflection, effectively enabling full-space functionality. Additionally, the bandwidth at half-intensity is used to evaluate the divergence of the beams. It is observed that the non-diffracting vortex beam exhibits excellent convergence when compared to the other two beams. Therefore, the designed non-diffracting beam metasurfaces show promise with respect to their future application in large-capacity and full-space terahertz communications.

## Data Availability

The data presented in this study are available in the manuscript.
